# Lived Experiences of Intensive Care Professional Nurses Caring for COVID-19 Patients in Private Hospitals in Gauteng, South Africa: A Phenomenological Study

**DOI:** 10.1155/2024/7225258

**Published:** 2024-04-25

**Authors:** Welhemina Molala, Charlene Downing

**Affiliations:** Department of Nursing, University of Johannesburg, Johannesburg, South Africa

## Abstract

**Aim:**

To explore and describe intensive care professional nurses' experiences caring for COVID-19 patients in private hospitals in Gauteng, South Africa.

**Introduction:**

Pandemics are unique forms of disasters characterised by adverse psychological symptoms and behaviours. Literature confirms a globally increased workload during pandemics, causing emotional exhaustion and poor concentration among healthcare workers. Moreover, high mortality rates are mentioned as a cause of moral distress and moral injury to healthcare workers. South Africa was unprepared for the COVID-19 pandemic, as evidenced by overcrowded hospitals, a lack of resources, and high mortality rates.

**Materials and Methods:**

A qualitative, phenomenological, exploratory, descriptive, and contextual research design was used. The five largest private hospitals from the same hospital group in Gauteng were chosen as they were admitting many COVID-19 patients. Fifteen participants were selected through purposeful sampling. Semistructured, in-depth, individual interviews were conducted and audio-recorded, and field notes were taken from April 2022 to December 2022. The interviews were transcribed verbatim and analysed using Giorgi's approach.

**Results:**

Three themes emerged: abrupt transition from normality to the COVID-19 pandemic; experienced isolation from family, community, and nursing management; and feelings of satisfaction and gratitude for teamwork and learning.

**Conclusions:**

It is essential for nurses' holistic care to be considered along with patients' holistic care. The findings in this study could drive healthcare institutions in South Africa to respond to nurses' health, care, and support needs. *Implication for Nursing Management*. Nursing management should consider shorter and fewer consecutive workdays for nurses to rest and restore their energy levels. Nurse management should also provide human caring by being visible to the nurses and communicating with them. Holistic self-care practices should be included in nurses' in-service training programmes.

## 1. Introduction

On 31 December 2019, the coronavirus disease 2019 (COVID-19) was reported to cause severe viral pneumonia in Wuhan, China [1]. The virus rapidly spread worldwide, resulting in a pandemic. COVID-19 is a complex respiratory disease that complicates acute respiratory distress syndrome (ARDS), and patients often require mechanical ventilation [2]. The transmission mode is human-to-human, and the virus is characterised by high transmission efficiency and the involvement of multiple organs [3].

South Africa's National Institute of Communicable Diseases (NICD) reported its first confirmed case on 5 March 2020 [4], and the president of South Africa declared a national disaster on 23 March 2020. The pandemic's rapid spread resulted in national lockdown restrictions on 27 March 2020. All citizens were forced to remain at home. Like other countries, healthcare workers in South Africa faced the unknown, highly contagious COVID-19 virus with inadequate resources and guidelines [5, 6]. An intensive care unit is mainly defined by highly trained nurses, doctors, and medical equipment that can assist in reversing organ dysfunction, such as mechanical ventilators and advanced physiological monitoring devices [7]. In South Africa, the training of the ICU nurse is regulated by R.212, and nurses must have the additional qualification “Medical Surgical: Critical Care Nursing” from the South African Nursing Council (SANC) [8]; a critically ill patient requires the skills of critical thinking and clinical judgment underpinned by scientific, biomedical, and technological knowledge [9]. However, in this study, professional nurses were unexpectedly transferred from the wards to ICUs without knowledge and skills of critically ill patients' care. Sufficient physical, emotional, and mental support is essential to promote nurses' ability to care for patients [10]. Yet, healthcare workers were under enormous stress and were physically, emotionally, and mentally challenged during the pandemic.

Cotton and Iro [11] affirmed that the world was unprepared for this pandemic, as evidenced by overcrowded hospitals, high mortality rates, and staff shortages. Ulrich, Rushton, and Grady [12] agree that a general lack of institutional preparedness for the volume of COVID-19 patients within hospital systems left frontline nurses physically and emotionally susceptible to a persistent sense of guilt and anxiety. Al-Dossary, Alamri, and Albaqawi [13] also confirm that COVID-19 challenged nurses due to the novelty of the disease, and a lack of information and training accelerated the rate of hospital infections. The unavailability of guidelines was also significantly associated with anxiety among nurses [14] who faced an unexpected situation and had no experience and skill to deal with COVID-19 [15].

The World Health Organization [1] highlighted a global shortage of six million nursing positions, and combined with the staff shortages during the pandemic, quarantine also made nursing care difficult. In addition, many healthcare workers in South Africa were infected with COVID-19, placing more strain on the remaining healthcare workers [16]. Carter and Notter [17] confirm that COVID-19 placed unprecedented pressure on critical care services, stretching resources beyond capacity. Increasing the number of beds in intensive care units (ICUs) to accommodate critically ill COVID-19 patients resulted in a higher workload for nurses [18]. Moreover, increasing ICU beds resulted in a shortage of equipment such as ventilators and high-flow nasal machines, and nurses became part of the ethical decision-making process in this context, reflecting on criteria such as beneficence, nonmaleficence, autonomy, and justice [19]. Eftekhar Ardebili et al. [20] indicated that participants reported a heavy workload, fast changes in the workplace, a loss of control over caregiving situations, feelings of helplessness, ineffectiveness in routine work, and the inadequacy of previous work experiences. Elhadi et al. [21] similarly indicated that a significant number of healthcare workers expressed low levels of awareness and preparedness regarding COVID-19, and their concern was that inadequate knowledge is a risk factor for disease transmission and can lead to low levels of care.

Mekonen et al. [14] pointed out that the unavailability of guidelines, fear of infecting family members, and having a chronic disease were significantly associated with nurses' anxiety. Nursing managers also described their management approach during the pandemic as novel and complex, and nurses were losing trust in them [22]. This same view is supported by Sperling [23], who explains that nursing care is extraordinarily stressful and demanding due to rapid changes in guidelines and regulations. Moreover, nurses' fear and a lack of technical skills and knowledge about COVID-19 were significant coefficients in their mental health [24]. The inability to assist patients was also demotivating and frightening for nursing staff. Robertson, Maposa, Somaroo, and Johnson [25] claim that healthcare workers' motivation and empathy are critical to effective and compassionate care. However, adverse mental health conditions were noted among healthcare workers exposed to COVID-19, and this affected their patient care. Witnessing unacceptable situations also causes individuals to react by blaming themselves, either for the choices they made or their inability to perform specific actions [26]. Moreover, inadequate knowledge in caring for patients with COVID-19 could result in moral injury, a form of psychological distress attributed to performing an action that contradicts one's own moral and ethical code, resulting in guilt, shame, and anger [27]. South Africa does not have clinical resource organisations supporting nurses' holistic care, such as the American Holistic Nurses Association, which encourages staff's well-being.

During the pandemic, healthcare workers were left alone due to restrictions to control the spread of the virus and expected to deal with patients' traumatic experiences and the unexpected loss of friends, family, and colleagues [28]. Said and El Shafel [29] also emphasise that inadequate emotional preparation among nurses dealing with death and dying is linked to occupational stressors. In addition, while working with COVID-19-positive patients, nurses wear heavy personal protective equipment (PPE) and often go six hours without using the bathroom, tolerating hunger and thirst. These working conditions affect the nurses' physical, physiological, emotional, and psychological health [30], and the PPE is considered inadequate and not user-friendly [23].

The COVID-19 pandemic has consistently been documented as negatively affecting nurses worldwide since the outbreak's start [31–33]. Several South African studies have explored nurses' experiences caring for COVID-19 patients during the pandemic. However, these studies were conducted in contexts and healthcare institutions that differ from this study. Those studies were carried out in primary healthcare facilities [34–37], and other studies included all healthcare workers: nurses, medical doctors, and allied health professionals [38]. To fill this gap, this study explores the lived experiences of professional nurses caring for COVID-19 ICU patients in private hospitals in Gauteng, South Africa.

## 2. Materials and Methods

The authors followed a descriptive qualitative research method to explore professional nurses' experiences caring for patients with COVID-19 in private hospitals in Gauteng, South Africa. The first author used the Consolidated Criteria for Reporting Qualitative Research (COREQ) guidelines in approaching and reporting on this qualitative study.

### 2.1. Study Design

A qualitative, exploratory, descriptive, and contextual research design was used in this study. In particular, we employed a phenomenological approach to inquiry, focusing on understanding individuals' lived experiences and the meaning they attribute to those experiences. Phenomenology aims to provide a rich description of human experiences as they are perceived and understood by individuals [39]. Through in-depth, individual interviews, we aimed to understand participants' lived experiences and the meaning they ascribed to them. This approach offers a detailed exploration of participants' perspectives within their natural contexts. Data analysis involves reducing and organising data and extrapolating meaning [40, 41]. It is also defined as a dynamic process of weaving together emerging themes and identifying key ideas or units of meaning and material acquired from the literature [42]. Giorgi's analysis method was used to uncover the meaning of the phenomenon as experienced by individuals through the identification of essential themes [43] (see Table 1).

#### 2.1.1. Settings

Data were collected at five private hospitals from the same private healthcare group. These private hospitals were chosen because they are the largest private hospitals in Gauteng and were admitting many COVID-19 patients during the pandemic because of their bed capacity. The distance between each hospital was eight to ten kilometres. The interview venue in each hospital was unique; boardrooms, clinical department offices, the night manager's office, and the infection control office were used for interviews. All five hospitals accommodated the first author in a way that ensured participants' privacy was protected and no disturbances occurred during interviews. Moreover, COVID-19 protocols were strictly followed during interviews, including wearing face masks and adhering to social distancing, and hand sanitisers were available in each interview room.

#### 2.1.2. Participants, Sample, and Sampling

In this study, the population was all professional nurses caring for COVID-19 patients in a private hospital in Gauteng. In this study, the researcher (first author) used purposive sampling. Grove and Gray [40] define purposive sampling as recruiting participants based on their knowledge, experience, or views related to the intended study. This study's purposive, convenient sample was professional nurses caring for ICU COVID-19 patients in five private hospitals of the same hospital group in Gauteng (*N*=15).

Inclusion criteria are the characteristics the participants must possess to be part of the target population [45]. This study's inclusion criteria were as follows: (a) all professional nurses in the chosen private hospitals caring for patients in COVID-19 ICUs during the pandemic; (b) the professional nurses had to be permanently employed at one of the five private hospitals, working day or night shifts in the COVID-19 ICUs; (c) participants had to be able to read and speak English; and (d) professional nurses who are working in the COVID-19 ICUs. Exclusion criteria are characteristics that restrict the population to a homogenous group of subjects [46]. This study's exclusion criteria were as follows: (a) professional nurses who did not work in the COVID-19 ICUs full time or those working as agency staff as they were often unavailable; (b) enrolled and auxiliary nurses working in the COVID-19 ICUs and general units, as they work under the direct supervision of professional nurses, and they were not assigned to nurse critically ill patients; and (c) professional nurses working in the general COVID-19 wards.

The first researcher, who is a deputy nurse manager, conducted personal recruitment; the researcher got expertise to conduct a phenomenological study. The researcher met with the hospital management of all five hospitals separately to explain the intended study in detail. All five hospitals were happy to be chosen for the proposed study, and they all agreed that the interviews could be conducted during shifts. The deputy nurse managers of each hospital were chosen as gatekeepers, as they are the most involved with staff; this view is supported by Aaltonen and Kivijärvi [47], who agree gatekeepers are professionals or individuals engaged in everyday practice.

The gatekeepers play a key role in ensuring researchers gain access to potential participants and sites for research [48]. The researcher left information letters with the gatekeepers in each hospital to be distributed to all professional nurses who worked in COVID-19 units during the pandemic. Fifteen participants contacted the researcher voluntarily to indicate their willingness to participate in the study [44]. The researcher contacted the gatekeepers to arrange a suitable venue after the participants agreed upon an interview date and time. The researcher was aware of possible coercion due to her position of power, but participation was entirely voluntary. Participants were also not compensated for their participation.

The sample consisted of five male and ten female participants who voluntarily agreed to participate. Informed written consent was obtained from each participant. Participants' ages ranged from 27 to 45; the mean age was 27. All the participants were professional nurses, and three had post-basic diplomas in medical, surgical, and critical care nursing [9]. Their nursing experience ranged from one to ten years. Participants' demographic information is summarised in Table 2.

### 2.2. Data Collection

This study explored the experiences of professional nurses working in COVID-19 units in Gauteng, South Africa, from the beginning of the first wave until the end of the fifth wave. However, data were only collected from the end of the fourth wave (April 2022) to the fifth wave (December 2022). All the participants had been working in COVID-19 units since the pandemic's outbreak in the country. The first author thoroughly explained the purpose of the study to participants before obtaining their informed consent. The interviews were conducted between 10h00 and 11h00 with the permission of the nursing service managers after the doctors' rounds and participants' morning routine to avoid disruptions to care. The gatekeepers also arranged for clinical facilitators to help, if needed, during the participants' interviews.

The researcher conducted semistructured individual interviews that lasted 45 to 60 minutes, using various communication skills to gather rich information, and recorded information using an audio recorder, observation skills, and field notes. The researcher gained experience in qualitative research during her master's degree study, and she gained expertise in phenomenological studies. The first interview was conducted in the presence of the second researcher, who has extensive research knowledge in qualitative research, a professor in nursing science. This interview was regarded as a pilot interview to determine the central question's effectiveness in gathering the required information based on the study's objective. The central open-ended question was as follows: “How is it for you to care for COVID-19-positive patients?” The pilot interview was successful, provided rich data, and was added as part of the data analysed for the main study. Each interview was transcribed verbatim on the day it was conducted and sent to the research supervisor for review. Data saturation was attained with the 15th interview, and the independent coder confirmed this saturation.

### 2.3. Data Analysis

Data analysis involves reducing and organising data and extrapolating meaning [49, 50]. To obtain a sense of the whole, the analysis starts by reading and rereading the data, looking at themes, emotions, and the unexpected, considering the overall picture [35]. Data were analysed for meaning using Giorgi's [43] approach, which involved the following steps: (a) the first author read the complete transcripts to get a sense of the whole and bracketed her preconceived knowledge and ideas regarding the phenomenon under study to focus on professional nurses' perspectives. The information was obtained and transcribed from in-depth individual interviews, audio recordings, and field notes. (b) The first author went back to the beginning of the transcriptions and reread them. This time, every time she experienced a transition in meaning within the participant's attitude, she made a mark on the transcription. These parts are called “meaning units.” (c) The authors transformed the data—still in the subject's words—into expressions that were more direct impressions of what the participant said. (d) The direct and psychologically more sensitive expressions were then reviewed, and with the help of free imaginative variation, an essential structure of the experience was formed. (e) The essential structure was then used to help clarify and interpret the raw data of the research.

The first author compiled, reanalysed, and interpreted the results. Data were manually coded using coloured pens to categorise the findings, identify significant patterns in all the interviews, and finally draw meaning. A discussion was held with the second author, and agreement was reached regarding the themes and subthemes that emerged, as differences only related to terminology and the phrasing of sentences. In May 2023, the first author also telephonically contacted the participants to confirm whether the data analysis was a true reflection of their meaning. None of the participants requested changes to the themes/subthemes.

### 2.4. Trustworthiness

Trustworthiness measures refer to the concepts adapted and promoted by Lincoln and Guba [51] to the essential framework for evaluating trustworthiness in qualitative research. Denzin and Lincoln [52] discuss the trustworthiness criteria: 1. credibility: (a) prolonged engagement was adhered to as data collection started on 23 April 2022 and was completed on 14 December 2022. (b) Triangulation occurred by the first author using multiple sources of data collection, including in-depth, individual interviews, observations, and field notes. (c) Peer debriefing with the research supervisor, a professor in nursing science with extensive knowledge in qualitative research studies, also ensued. The supervisor reviewed and challenged the analysis to promote the rigour and credibility of the data analysis. (d) Member checking: collected data were verified with the participants, who confirmed that the findings reflected their feelings and experiences. 2. Transferability: (a) purposive sampling was used, and a description of the participants' demographic profile and the setting was provided. (b) The research methodology was described in detail. 3. Dependability was heightened in the discussion of the results. 4. Confirmability: adequate and relevant references were used, and there are retrievable, well-organised collected data.

In addition, the first author wrote reflexive notes, indicating a lot of introspection and internal examination to explore her feelings, experiences, and biases, which were bracketed to enhance objectivity. As defined by Polit and Beck [53], bracketing is a method some qualitative researchers used to reduce potential taints caused by preconceptions. This study reminded the researcher of her experiences of being infected with COVID-19, and she found it challenging not to discuss these experiences with participants but to pause, take a drink of water, and refocus on the participant.

### 2.5. Ethical Approval Details

The study was approved by the Faculty of Health Sciences of the University of Johannesburg's Higher Degrees Committee (HDC-01-96-2021), the Faculty of Health Sciences Ethics Committee (REC-1252-2021), and the research committee of a private hospital group where the participants were employed (UNIV-2021-0054).

## 3. Results

The sample consisted of five male and ten female professional nurses aged 27 to 45; the mean age was 27. Three main themes emerged, and these were further subdivided into subthemes. The narrative that follows describes the qualitative themes and offers quotations demonstrating how professional nurses' physical, mental, emotional, and social health was affected during the pandemic.

### 3.1. Theme 1: Abrupt Transition from Normality to the COVID-19 Pandemic

Participants described the COVID-19 as overwhelmed, overworked, physically and mentally exhausted, and faced high mortality rates, leaving them feeling overwhelmed and overworked. Professional nurses' unexpected transfers from general wards to ICUs to care for very sick patients also created significant frustration for ICU professional nurses. They had to teach their colleagues from the general wards how to nurse very sick patients on ventilators. Moreover, professional nurses from the general wards were frustrated because they had to learn fast.

#### 3.1.1. Subtheme 1.1: Participants Felt Overwhelmed, Overworked, Physically and Mentally Exhausted, and Faced High Mortality Rates

The participants also mentioned that there was enormous pressure on them as they did not know about COVID-19. Participants feared possible errors and were held accountable even though they were not the primary nurses because professional nurses with a lack of ICU experience from the general wards were working under their direct supervision. In normal situations, the shift leader supervises and supports the team to ensure safe patient care. The shift leader is thus held accountable for the team and their actions. Participant 5 shared: “*Nurses from the ward didn't know how to nurse very sick patients on the high flow machines, Continuous positive airway pressure (CPAP) machines and ventilators. We had to teach them quickly the basic things like trouble shooting when ventilator alarms and the most important things, not to ignore the alarms. The patients were very sick, more pressure on us. They were working under direct supervision of us; we were responsible and accountable for their patients and our patients.*” During the pandemic, no one was assigned to shift leading, and ICU staff already taking care of more than three critically ill patients at a time were simultaneously responsible for staff from the general ward assigned next to them.

The shortage of nurses resulted in prolonged shifts and increased professional nurses' duties. Extended shifts led to extreme physical and mental exhaustion. Participant 8 said: “*shortage of staff affected us negatively. Most of the staff members were infected. Nursing three patients and sometimes you find us that you are the only one who is ICU trained, accountability is on your shoulders.*” Participant 1 shared his sadness: “*We worked ten days or more straights shift to cover the units. There was a time where I had to look after four High care patients alone due to shortage of staff.*” Participant 11 mentioned a similar experience: “*Yes it was hell. We planned that we would eat before going in and use the bathroom as we know that we will wait for 6 hours to drink, eat or use the bathroom. Going out and leaving your patients with your colleague who is already battling with more than 5 patients was not easy. We opted to go and eat quickly and come back, not to spend an hour break.*”

Participants were unhappy with the rapid changes in the COVID-19 policies. “*We felt that management was failing us, changing protocols many times but not coming to us to hear our problems*” (*Participant 7*). Participant 4 mentioned the same challenge: “*Management were changing protocols daily or twice a day, sometimes we were not getting those communications only after work when you switch on your phones you will get lots of forwarded messages from our unit manager, received from the management.*”

Participants witnessed many deaths and suffered moral distress, which resulted in moral injury. Participant 3 reportedly experienced significant distress as a result of the multiple deaths they faced during the pandemic: “*second wave was bad in that most people were dying, arriving at home, you just have to bath and sleep. When you are sleeping, your mind is still going through all what happened during the day, you cannot sleep. Most of us were depressed due to lots of death.*” Participant 9 said: “*You hear your colleague crying after being called to the phone, just to tell her that somebody in their family died. She will cry and go out for 30 minutes and come back to look after her patients. It was a norm that you will attend the funeral to only your immediate family, not the uncles or cousins. It was bad. I am from extended family; we are too close to uncles and cousins, but we couldn't bury them.*”

Participants also indicated that the extra burden of patient care led to self-neglect, and they had to sacrifice their own holistic needs. Participants put the patients first and neglected their basic needs; they had to wear protective gear for up to six hours at a time due to the shortage of PPE and staff. Consequently, participants were facing dehydration and discomfort. Participant 2 explained: “*we were in the ward for 6 hrs. In that six hours we could not eat, or drink anything. We were sweating to the point that we were thirsty. We kept on working because our patients were very sick.*”

### 3.2. Theme 2: Experienced Isolation from Family, Community, and Nursing Management

#### 3.2.1. Subtheme 2.1: Participants Experienced Stigma from the Community and Isolation from Family

The participants indicated that their relationships with friends, families, and the community changed during the pandemic. Some community members labelled them as having COVID-19. The participants said they were isolated and often avoided. Participant 5 shared: “*…when I am off, I will stay in my flat because people in our building were assuming that if you are a nurse, you got Covid. You can see the way they were looking at me and suddenly avoiding me.*” A similar perception of social isolation was shared by Participant 2: “*I have lost my cousin, my uncle's son. I couldn't go and bury him. After a week, his wife died as well, and I couldn't go and bury her. We were so close; we were like twins. Same age as me. So, my uncle is angry with me, he even told my parents that I must not come to his house. I am from the rural area, people there will never understand.*”

#### 3.2.2. Subtheme 2.2: Participants Experienced Isolation and a Lack of Support from Nursing Management

Most participants felt management should have done more to provide sufficient PPE to protect the staff. They also concurred that the information should have been more consistent and clearer. The participants mentioned that news of the deaths of colleagues caused them tremendous psychological harm, and management did not comfort them. This increased their fear that they were facing the danger alone, and they worried that it might happen to them. Many participants ultimately mentioned a lack of support from management during the interviews. Participant 4 said: “*There was a shortage of PPE as the hospital was getting full. Zapping of gowns and N95. We were not even sure if they are 100% safe. We felt the hospital is gambling with our lives, moreover they were not even coming inside just to say thank you. Only people from outside on TVs and radios that were acknowledging our hard working. It is very sad.*”

### 3.3. Theme 3: Feelings of Satisfaction and Gratitude for Teamwork and Learning

#### 3.3.1. Subtheme 3.1: Participants Expressed That COVID-19 Gave Them Learning Opportunities and Empowered Them to Mature Professionally

The regular ICU staff described being grateful for the support they received from colleagues from other departments. Similarly, nurses who were transferred to the ICU shared their gratitude for the support from the ICU nurses. Participants mentioned that their resilience was enhanced by shouldering the COVID-19 burden with their colleagues. Participant 5 explained: “*…it was not easy, but we were a good team of nurses and doctors. We supported one another. We told ourselves that we need to be there for our patient.*”

#### 3.3.2. Subtheme 3.2: Participants Expressed Feelings of Satisfaction

The participants expressed their pleasure at seeing patients recover and return home; it was a moment of joy for them. This was often described as a victory over the disease and gave the professional nurses hope for other patients and the strength to continue their work. Additionally, the participating nurses described many emotional moments, for example, when patients could communicate with their relatives for the first time in weeks. Participant 10 reflected: “*It was hectic, but we were grateful if we extubate a patient and see them recovering well, it was very pleasing. Discharging patient home was a bonus, we will be so excited. Patients will phone us and update them about their progress, we were also doing the follow up, they were concerned about us. They were so grateful.*”

Themes, subthemes, and participants' quotes are summarised in Table 1.

## 4. Discussion

The results of this study revealed that professional nurses experienced posttraumatic stress due to multiple stressors such as a shortage of staff, inadequate knowledge of COVID-19, high mortality rate, isolation from family, and stigma from the community. Moreover, a lack of support from nursing management was emphasised. The participants shared their negative experiences linked to physical and mental exhaustion, sleepless nights, fear, depression, and a lack of holistic self-care practices. Although the nurses mentioned some negative experiences, they expressed satisfaction and gratitude for their team's efforts (Table 1, [48]).

*Professional nurses described the COVID-19 pandemic as an abrupt transition from normality to the COVID-19 pandemic*. It was an unexpected chaotic disruption in the care of patients due to the overflowing of patients in the hospitals despite a gross shortage of staff. Moreover, most of these COVID-19 patients were critically ill in the ICUs. This resulted in rapid changes in working structure and more responsibilities as the professional nurses with a lack of knowledge and skills in nursing a critically ill patient were moved from the wards to the ICUs. The South African Nursing Council Competencies for Critical Care Nurses' Nursing Act (Act No. 33 of 2005) [9] suggests that patient care in ICUs should be comprehensive critical care. Nursing practice in South Africa is governed by the R767 regulations, which outline the acts and omissions for which the SANC can take disciplinary action (Nursing Act No. 33 of 2005) [54], such as failing to maintain a patient's health status. The nurse-to-patient ratio in the ICUs is 1 : 1; however, in this study, the professional nurses mentioned that they were nursing three critically ill patients. Participants shared that they worked ten days or more straight shifts to cover the unit, which is against the South Africa Basic Conditions of Employment Act [55], which states that an employer may not require or permit an employee to work more than 45 hours in any week.

*Professional nurses mentioned that they were overwhelmed, overworked, and physically and mentally exhausted.* Similar findings were mentioned by Yunitri, Chu, Kang, Jen, Pien, Tsai, Kamil, and Chou [56] that hospitals and clinics overcrowded with COVID-19 patients left health professionals with no time for rest, impacting their psychological well-being. This is asserted by Liu et al. [57] that intensive work during the COVID-19 pandemic drained healthcare providers physically and emotionally. Alquwez [58] mentioned that as nurses deal with a terrible pandemic and exhausting work experiences, well-being at work is critical and needs to be emphasised. Inocian, Cruz, Saeed Alshehry, Alshamlani, Ignacio, and Tumala [59] also mentioned that nurses' work experiences and work conditions during this pandemic adversely affected their professional quality of life. Although the South African Nursing Council's (SANC) Nurses Rights [60] states that nurses have the right to a safe working environment that is compatible with efficient patient care and is equipped with at least the minimum physical, material, and personnel requirements; however, the working environment was not physically and mentally safe for the participants in this study.

*During the interviews, professional nurses expressed frustration and stress regarding the frequent changes in COVID-19 protocols and policies*. Catania et al. [61] wrote that the main challenges their participants raised were the constant change in care management guidelines and treatment protocols, which resulted in nurses providing care in uncertain conditions. LoGiudice and Bartos [62] also shared that the nurses in their study expressed that their stress was heightened because their hospital's protocols changed daily. There was a lot of confusion from the administration with changing guidelines, directions, and endless questions. Persistent changes in protocols and policies were also mentioned by Firouzkouhi et al. [63], stating that despite nurses' crucial role in public health in critical situations such as pandemics, they face certain obstacles in managing events when there are no predefined guidelines or protocols. Irrespective of the frustrations and confusions due to unclear, inconsistent guidelines, professional nurses are obliged to adhere to the South African Nursing Council's (SANC) Code of Ethics [64], which states that nurses are required to demonstrate the art of nurturing by both applying professional competencies and positive emotions.

*Professional nurses faced moral distress and moral injury due to high mortality rate.* The participants expressed that they were mentally affected due to the high mortality rate, and some could not sleep as a result. Their distress was also related to patients dying alone due to the visitor restrictions that were imposed during the pandemic. Lake et al. [65] reported that the most distressing situations for professional nurses included caring for patients dying without family present. Robinson and Stinson [65] similarly concurred that their participants were stressed at seeing patients dying without their loved ones by their side. Patients with COVID-19 faced lonely deaths, and family members were not allowed to be present with their loved ones [66]. Castaldo Lusignani Papini Eleuteri and Matarese [67] stated that nurses consequently suffered emotionally and felt inadequate to respond to the needs of many dying patients during the COVID-19 pandemic. Several studies concluded that posttraumatic stress disorder is a major concern in health institutions [68]. Spilg et al. [69] concurred that healthcare workers caring for COVID-19 patients showed signs of severe moral distress, anxiety, and depression. Hossain and Clatty [70] stated that the stress nurses face would create moral distress and have a lasting impact; this is why the term “moral injury” is most suited in the context of COVID-19. This view is supported by Al Maqbali [71], who claims nurses experienced sleep disturbance related to increases in stress, anxiety, depression, high workloads, fear, pressure, and helplessness during the pandemic; to care responsibly for patients, especially as they practice in settings with more complex needs, nurses need to feel healthy, well, and supported. In this study, the participants were not offered any form of psychoemotional support.

*Participants also indicated that the extra burden of patient care led to self-neglect, and they had to sacrifice their holistic needs*. Participants' needs were compromised, and they shared that they kept working in unfavourable conditions without eating or drinking for extended periods. During the interviews, the professional nurses shared that they were wearing their PPE for up to six hours before they could go out for a break, leading to sweating and itching. The South African Government's Batho Pele Principles,1977 [72], “putting other people first,” and the South African Nursing Council's (SANC) Pledge of Service [73] were applied by professional nurses during the COVID-19 pandemic, putting patients first and neglecting their basic needs. Abiakam, Worsley, Jayabal, Mitchell, Jones, Fletcher, Spratt, and Bader [74] indicated that wearing PPE for more than four consecutive hours led to redness of the cheeks, dry mouth, redness of the nose bridge, and redness of the ears due to N95 masks, dryness of the mouth when wearing surgical masks, skin dryness, sweating, and redness from wearing gloves, headaches from wearing goggles/face shields, and sweating when wearing overalls or a gown. Harris, McLeod, and Titler [75] similarly stated that the lack of breaks, including meal and bathroom breaks, affected their participants' physical health. Wearing PPE for a long time exposed nurses to physical health complications such as pressure injuries, dermatitis, dehydration, and headaches [76, 77]. Every employer shall provide and maintain, as far as is reasonably practicable, a working environment that is safe and without risk to the health of his employees, according to Occupational Health and Safety Act 85 of 1993 [78].

*Professional nurses experienced social isolation from their family and friends.* Professional nurses shared that they had a fear of transmitting the virus to them and decided to isolate themselves to protect families and friends. However, it was negatively affecting their mental well-being. Many nurses felt social rejection, the closeness of the relationship, a sense of social rejection, and a high level of loneliness and depression [79]. Many nurses had quarantined themselves from their families and others to prevent the risk of transmission, which had exacerbated their feelings of anxiety, stress, and social isolation [80]. Çelik et al. [81] also revealed that nurses felt pain because the pandemic process had separated them from their families and children.

*Isolation from the community affected the professional nurses*' *mental well-being negatively*. Some professional nurses indicated that their relationships with friends, families, and community changed during the pandemic. Much of this was related to the fear of transmitting the virus to them, and some community members were labelling them as having COVID-19. Nurses who cared for patients diagnosed with COVID-19 experienced stigma and were labelled “COVID nurses” [82]. Asa et al. [83] shared that nurses were distressed due to stigma and discrimination against nursing COVID-19 patients. Ramaci et al. [84] also indicated that during the COVID-19 pandemic, healthcare workers were facing stigma, resulting in psychological distress. In South Africa, a nurturing of humanism called “Ubuntu,” meaning a human being is a human being through the otherness of other human beings, is supported throughout the country. However, this study revealed that the community did not support nurses during the pandemic.

*Moreover, most participants mentioned a lack of support from nursing management and their invisibility during these extremely challenging times*. Participants expressed that they were facing the danger of the new virus alone, and nursing management was not visible. Dawood, Tomita, and Ramlall [85] highlighted high levels of depression, anxiety, and stress, combined with poor perceptions of employer support, illustrating the need to identify and address this population's psychosocial support needs. During pandemics, healthcare organisations should maintain clear, fluid, and regular communication with nursing staff, which could help increase staff members' confidence and sense of control [86]. It was also confirmed by Joo and Liu [87] that nurses did not receive adequate support from hospitals and the healthcare system and lacked the necessary protective equipment, such as masks and hand sanitiser, to ensure the safety of healthcare workers. AL‐Abrrow et al. [88] suggested that there is an excellent need for managerial interventions, for example, support, appreciation, and recognition, to help healthcare workers feel valued in their work. Inadequate leadership support was also attributed to a lack of support for the healthcare team, including inadequate staffing and supplies [89]. Managers should thus respond appropriately to decrease nurses' COVID-19-related phobias by educating, counselling, or providing psychotherapy and work-life balance strategies.

*Participants expressed the feelings of satisfaction and gratitude for teamwork and learning.* This view is consistent with other studies, reflecting that healthcare workers' ability to support their colleagues creates a growth environment [90]. Veitch and Richardson [91] pointed out that coworker support benefits nurses' mental well-being during times of crisis. Nurses often put the team's needs before their own and hold a cultural ideal of team loyalty; they often work even when sick so they do not let the team down [92].

*Participants mentioned that COVID-19 empowered them to mature professionally*. In this study, professional nurses demonstrated resiliency and professionalism. Participants verbalised that they had grown professionally and learned a lot; this finding is consistent with Molala and Downing's [93] findings that their participants were eager to learn and achieve professional growth despite the challenges of working in a new environment. Although the provision of care led to physical and psychological distress among nurses, based on their commitment and professional obligations, this new experience also led to personal satisfaction [94]. Clinical nurses gained significant experience during the pandemic, positively affecting their readiness for future pandemics [95]. Alcalá-Albert et al. [96] also shared that their participants mentioned that clinical practice provided them with additional caregiving knowledge and techniques that were previously unknown to them. LoGiudice and Bartos [67] concurred that their participants experienced professional growth during the COVID-19 pandemic and felt they could deal with difficult situations.

*When patients survived and were discharged, feelings of satisfaction felt like an achievement for professional nurses.* Additionally, the participating nurses described many emotional moments, such as when patients could communicate with their relatives for the first time in weeks. Positive feelings when patients recover are embedded in Watson's theory as one of the Caritas, humanistic-altruistic value systems. Positive experiences were also mentioned by Taheri-Ezbarami et al. [97], who determined that caring for patients with COVID-19 satisfied nurses' needs, such as altruism and love for others, and it ultimately led to the formation of a sense of satisfaction in providing care.

## 5. Conclusions

This study revealed that psychosocial, mental, and physical care are paramount to professional nurses' ability to deliver holistic care to themselves and their patients. The authors hope this study's findings will help healthcare organisations develop new strategies and policies to support and prepare nurses for future pandemics and outbreaks. Moreover, professional nurses' holistic self-care should ultimately be facilitated in nursing. Unfortunately, South Africa does not have clinical resource organisations that support nurses' holistic care, such as the American Holistic Nurses Association, which supports the well-being of nurses. The authors hope the nurses' voices will be heard, and awareness of the need to promote their mental health will be created. This information could drive healthcare institutions to plan for future pandemics and respond to nurses' health, care, and support needs [98].

## 6. Implication of the Study

To make nurses feel valued in their jobs, nursing management must provide support, appreciation, and recognition. It is recommended that nursing management consider shorter and fewer consecutive workdays for nurses so they can rest and restore their energy levels. Nursing management should provide human caring by being visible to the nurses and communicating with them continuously. Holistic self-care should be included in nurses' in-service training programmes. It is also essential that health institutions offer well-being and mental health support to their employees.

## 7. Recommendations for Future Research

Using the findings of this study, a quantitative study could be conducted to generate a questionnaire and distribute it to public and private hospitals; these findings could emphasise the need to support nurses' well-being. The study concurred on the need for a model to facilitate nurses' holistic care; this can lead to improved patient care and quality of life for nurses.

## 8. Study Limitations

Fifteen nurses were recruited from five private hospitals but none from state hospitals for this study. Therefore, the findings cannot be generalised to other populations. The study was conducted from the end of the fourth wave to the end of the fifth wave of the pandemic; if it had been conducted during the first wave, more experiences might have been shared as they happened. The first author is a deputy nurse manager, and although the study was not conducted in the hospital where she works, it is the same hospital group; thus, participants might not have felt comfortable sharing some of their experiences. Nursing categories such as enrolled and auxiliary nurses were excluded, limiting the overview of nurses' experiences during COVID-19.

## Figures and Tables

**Table 1:** Themes, subthemes, and quotes from participants [44].

Subthemes	Categories	Quotations
*Theme 1: abrupt transition from normality to the COVID-19 pandemic*		
(1.1) Participants felt overwhelmed, overworked, physically and mentally exhausted, and faced high mortality rates	Rapid changes in working structure, COVID-19 policies, and more responsibilities	Participant 7: *“We felt that management was failing us, changing protocols many times but not coming to us to hear our problems”*
	Participants were overworked due to a shortage of staff	Participant 2: *“We were not prepared; we did not know the signs and symptoms of it and how it spread from one person to another. We were all scared, and we were overworked”*
	Participants faced moral distress and moral injury	Participant 8*: “…shortage of staff affected us negatively. Patients were left with us; no visitors were allowed. Most of the staff members were infected. Nursing three patients and sometimes you find us that you are the only one who is ICU trained. Most of the staff never nurse a ventilated patient” Participant 1 shared his sadness: “We worked ten days or more straight shift to cover the ward”*
	Participants witnessed an increased death rate	Participant 4: *“We are still not recovered emotionally from seeing lots of people dying. We have lost so many patients”*
	The extra burden of patient care led to self-neglect, and nurses sacrificed their own holistic needs	Participant 2: *“We were in the ward for 6 hrs. In that six hour we couldn't eat, or drink anything. We were sweating to the point that we were thirsty. We kept on working because our patients were very sick”*

*Theme 2: experienced isolation from family, community, and nursing management*		
(2.1) Participants experienced stigma from the community and isolation from family	Participants sacrificed seeing their families and friends and suffered loneliness	Participant 3: *“You arrived at home after a hectic day, you are tired physically and emotionally, you can't share with your family because you are scared that you might infect them, you isolate yourself”*
	Participants experienced stigma from the community	Participant 5: *“…when I am off, I will stay in my flat because people in our building were assuming that if you are a nurse, you got Covid. You can see the way they were looking at me and suddenly avoiding me”*
(2.2) Participants mentioned a lack of support from nursing management	Participants shared that nursing management did not physically or emotionally support them during extremely challenging times	Participant 4: *“Nursing management was not even coming inside just to say thank you. Only people from outside on TVs and radios that were acknowledging our hard working. it is very sad”*

*Theme 3: feelings of satisfaction and gratitude for teamwork and learning*		
(3.1) Participants expressed that COVID-19 gave them learning opportunities and empowered them to mature professionally	Participants experienced very positive team support	Participant 7: *“Jaa, we were good team there were some instances when we talk about the incidents that happened and how to improve ourselves. Yes, and after talking we were feeling better. We were praying every morning before starting the routine and it was helping”* Participant 1: *“I have learned a lot. After so much that I have learned, I want to do a degree in Nursing. Nursing COVID-19 patients has encouraged me to study more. I have realized that I need more knowledge”*
(3.2) Participants expressed feelings of satisfaction	Participants shared their joy when patients recovered	Participant 4: *“It was hectic, but we were grateful if we extubate a patient and see them recovering well, it was very pleasing. Discharging patient home was a bonus, we will be so excited. Patients will phone us and update them about their progress, we were also doing the follow up, they were concerned about us. They were so grateful”*

**Table 2:** Summary of participants' demographic information [44].

Participant	Gender	Age	Qualification	Post-basic qualification	Years of experience as a professional nurse	Department working prior to COVID-19
1	Male	27	Professional nurse		1	Surgical ward
2	Female	29	Professional nurse		1	Medical ward
3	Male	41	Professional nurse		4	Orthopaedic ward
4	Female	33	Professional nurse		10	Oncology
5	Female	29	Professional nurse		2	Surgical
6	Female	45	Professional nurse		4	High care unit
7	Female	31	Professional nurse	“Diploma in Medical Surgical: Critical Care Nursing” (SANC, 1993: R.212), March 2017	6	Intensive care unit
8	Female	38	Professional nurse		3	Surgical ward
9	Female	29	Professional nurse		3	Medical ward
10	Male	45	Professional nurse	“Diploma in Medical Surgical: Critical Care Nursing” (SANC, 1993: R.212), March 2017	10	Intensive care unit
11	Female	32	Professional nurse		3	Orthopaedic ward
12	Male	41	Professional nurse		1	High care unit
13	Male	28	Professional nurse		3	Surgical ward
14	Female	45	Professional nurse		6	Medical ward
15	Female	31	Professional nurse	“Diploma in Medical Surgical: Critical Care Nursing” (SANC, 1993: R.212), March 2017	8	Intensive care unit

## Data Availability

The data used to support the findings of this study are available from the corresponding author upon reasonable request.
